# A Diagnostic Nomogram Based on ^18^F-FDG PET/CT for Bone Metastasis of Gastric Cancer

**DOI:** 10.3389/fcell.2021.783466

**Published:** 2021-12-14

**Authors:** Chunhui Wu, Xiaoping Lin, Zhoulei Li, Zhifeng Chen, Wenhui Xie, Xiangsong Zhang, Xiaoyan Wang

**Affiliations:** ^1^ Department of Nuclear Medicine, The First Affiliated Hospital of Sun Yat-sen University, Guangzhou, China; ^2^ Department of Nuclear Medicine, Sun Yat-sen University Cancer Center, Guangzhou, China

**Keywords:** gastric cancer, bone metastasis, ^18^F-FDG PET/CT, diagnostic model, clinical factor

## Abstract

**Purpose:** To develop an effective diagnostic model for bone metastasis of gastric cancer by combining ^18^F-FDG PET/CT and clinical data.

**Materials and Methods:** A total of 212 gastric cancer patients with abnormal bone imaging scans based on ^18^F-FDG PET/CT were retrospectively enrolled between September 2009 and March 2020. Risk factors for bone metastasis of gastric cancer were identified by multivariate logistic regression analysis and used to create a nomogram. The performance of the nomogram was evaluated by using receiver operating characteristic curves and calibration plots.

**Results:** The diagnostic power of the binary logistic regression model incorporating skeleton-related symptoms, anemia, the SUVmax of bone lesions, bone changes, the location of bone lesions, ALP, LDH, CEA, and CA19-9 was significantly higher than that of the model using only clinical factors (*p* = 0.008). The diagnostic model for bone metastasis of gastric cancer using a combination of clinical and imaging data showed an appropriate goodness of fit according to a calibration test (*p* = 0.294) and good discriminating ability (AUC = 0.925).

**Conclusions:** The diagnostic model combined with the ^18^F-FDG PET/CT findings and clinical data showed a better diagnosis performance for bone metastasis of gastric cancer than the other studied models. Compared with the model using clinical factors alone, the additional ^18^F-FDG PET/CT findings could improve the diagnostic efficacy of identifying bone metastases in gastric cancer.

## Introduction

Although the morbidity and mortality rates of gastric cancer have declined in developed countries, this type of cancer is still one of the most common malignant tumors worldwide and the fourth leading cause of death among cancer-related diseases globally (Sung et al., 2021). The incidence of bone metastasis (BM) from gastric cancer is very low, with values ranging from 0.9 to 3.8% (Mikami et al., 2017; Wen et al., 2019; Imura et al., 2020). However, autopsy findings suggest an incidence of gastric cancer bone metastasis of 13.4–15.9% (Turkoz et al., 2014), indicating that the rate of BM from gastric cancer (GC) is underestimated.

Patients suffering from GC with coinstantaneous BM usually have a poor prognosis. Their median survival time ranges from 4 to 9 months (Mikami et al., 2017; Qiu et al., 2018; Imura et al., 2020) after the diagnosis of bone metastasis. The 1-year survival rate is only 9.7% (Nakamura et al., 2014). The overall survival rate of patients with GC who received chemotherapy before the diagnosis of BM but without palliative chemotherapy after being diagnosed with BM is low (Wen et al., 2019). Moreover, chemotherapy and bone-modifying agents could significantly improve the median survival time (Imura et al., 2020). Therefore, early detection of BM in patients with GC is important for determining a suitable treatment plan and for prognosis.

Studies have shown that skeleton-related events (SREs), alkaline phosphatase (ALP), lactate dehydrogenase (LDH), carcinoembryonic antigen (CEA), and carbohydrate antigen 19-9 (CA19-9) are independent prognostic factors for patients with BM from GC (Park et al., 2011; Turkoz et al., 2014; Lim et al., 2016; Wen et al., 2019; Imura et al., 2020). ALP is recognized as a biochemical marker for the presence of BM in GC, but its use as a diagnostic tool still shows inadequate performance (Lim et al., 2016). Several studies demonstrated that the combination of clinical indicators such as ALP, CEA, and CA19-9 showed better diagnostic results than using a biochemical marker alone to detect BM in patients with GC (WANG and QIN, 2020). However, this model has not been rigorously validated.

Positron emission tomography/computed tomography with 2-fluoro2-deoxy-d-glucose (^18^F-FDG PET/CT) was reported to be more effective for the diagnosis of BM from GC in the initial staging workup than whole-body bone scanning and CT (Ma et al., 2013; Kawanaka et al., 2016). To our knowledge, no studies have reported a combination of PET/CT and biochemical markers to diagnose GC with BM. In the present study, we explored an effective diagnostic model combining ^18^F-FDG PET/CT and clinical data for BM in GC and assessed its diagnostic performance.

## Methods and Materials

### Patients

The data were recorded from patients with gastric cancer who underwent ^18^F-FDG PET/CT examinations in the First Affiliated Hospital of Sun Yat-sen University from September 2009 to March 2020. The inclusion criteria were as follows: 1) GC was pathologically confirmed; 2) morphological changes in bone or FDG-avid nodes in bones were revealed on ^18^F-FDG PET/CT. Patients with coexisting malignant tumors were excluded. As a result, data from 212 patients were retrospectively analyzed. The following criteria were used as references to confirm bone metastasis: 1) histopathological examinations; 2) one or more similar bone lesions found in the imaging examination, and follow-up examinations for more than 6 months showing a significant progression of bone lesions.

### Clinical and Pathological Data

Data from all 212 patients, including age, sex, skeleton-related symptoms, anemia, pathological types of GC (adenocarcinoma/signet ring cell carcinoma/mixed carcinoma/carcinoid), biomarkers (ALP, LDH, CEA, CA19-9), and metastasis characteristics (synchronous/metachronous metastasis), were collected from the Electronic Medical Record system. Patients who had bone metastasis at the time of diagnosis of gastric cancer were defined as synchronous bone metastasis.

### ^18^F-FDG PET/CT Image Acquisition

All patients fasted for at least 6 h before the administration of PET/CT using a GEMINI GXL PET/CT scanner (Philips, Amsterdam, Netherlands; Discovery STE; GE Healthcare, Milwaukee, WI, United States). A dose of 5.18 MBq (0.14 mCi)/kg ^18^F-FDG was administered intravenously, and then image acquisition was performed 60 min after the injection. First, a noncontrast CT scan of a slice thickness of 3 mm and a pitch of 1 and a matrix of 512 × 512 pixels was acquired, followed by PET acquisition, which was performed with a matrix of 128 × 128 pixels and a slice thickness of 1.5 mm before the imaging data were corrected for photon attenuation, decay and collection time. The ordered subsets expectation maximization method was applied in the reconstruction and fusion of the PET images (Wang et al., 2013).

### PET/CT Imaging Data

All PET/CT images were interpreted by two experienced nuclear medicine physicians to reach an agreement in terms of the gastric lesions, bone changes and bone lesions. To evaluate the tracer uptake of the lesion by semi-quantitative analysis, the region of interest (ROI) was placed on the entire lesion from the transverse PET image. The maximum standardized uptake value (SUVmax) was calculated. The imaging data, including the SUVmax of gastric lesions and bone lesions as well as bone changes (osteolytic changes/osteogenic changes/mixed changes/no obvious bone changes), were recorded.

### Statistical Analysis

Statistical analyses were performed using *R* 4.0.3 (The *R* Core Team, *R* Foundation for Statistical Computing, Vienna, Austria) running on *R Studio* 1.3.1093 (*R Studio* Team, *R Studio* Inc. Boston, MA, United States).

Categorical data are shown as frequencies with percentages, while quantitative data complying to normal distribution are expressed as the mean ± standard deviation (x ± *s*). The quantitative data not complying to a normal distribution are expressed as the median (quartile) [M (P25, P75)].

Then, all quantitative data were converted into categorical variables according to the quartile. Univariate regression analysis was performed to assess the factors related to gastric cancer with bone metastasis results as dependent variables and to screen the main factors (*p* < 0.05). The total data set was randomly divided into the training set for constructing the model and the validation set data for evaluating the diagnostic performance of the model according to the ratio of 8:2. The selected factors with different combinations were then classified into three groups of multivariable models by using training data. These three groups of models were generated and included different data: clinical factors, ^18^F-FDG PET/CT imaging factors, and additional ^18^F-FDG PET/CT imaging factors with the model generated from the clinical factors. Subsequently, 10-fold cross-validation was conducted, and three final models (clinical factors, ^18^F-FDG PET/CT imaging factors, both clinical and imaging data) were selected with smaller classification rates. Selected variables in logistic regression modeling for the training set were as follows: clinical factors included SRE, anemia, ALP, CEA, CA199, LDH; ^18^F-FDG PET/CT imaging factors included SUVmax of bone lesions, bone changes, location of bone lesions; ^18^F-FDG PET/CT imaging factors combined with the model generated from the clinical factors included skeleton-related events, anemia, ALP, CEA, CA199, LDH, SUVmax of bone lesions, bone changes, and the location of bone lesions.

Receiver operating characteristic (ROC) curves and calibration curves were plotted to evaluate the discriminating ability and predictive ability, respectively. The predictive ability was reflected by comparing observed probabilities with model-predicted probabilities of the three models by using training set data, validation data and total set data (Han et al., 2016; Hoshino et al., 2018; Park and Han, 2018). The AUC was compared using the Delong method. Finally, a nomogram for diagnosing bone metastasis of gastric cancer was created based on the binary logistic regression model with good discriminating ability and predictive ability.

## Results

### Patients and Lesion Characteristics

A total of 212 patients with GC who met the inclusion criteria were included in the study; 85 patients (40.09%) were diagnosed with BM, and 127 patients (59.91%) were diagnosed without BM (Table 1). From our data, patients with BM (52 ± 16) were younger than those without BM (59 ± 16). Only 24 patients (28.24%) with BM showed skeletal-related events (SREs), such as bone pain and pathological fractures. Anemia was detected in 62.26 and 60.00% of patients with BM and without BM, respectively. Adenocarcinoma (85.85%), neuroendocrine tumors (8.02%), adenocarcinoma mixed with signet ring cell carcinoma (4.72%), and signet ring cell carcinoma (1.41%) were the four most common pathological types of gastric cancer.

**TABLE 1:** Characteristics of all patients.

	Total	BM^a^	NBM^b^
Number of patients	212	85 (40.09%)	127 (59.91%)
Age (Mean ± SD)	56 ± 16	52 ± 16	59 ± 16
Gender			
Male	123 (58.02%)	48 (56.47%)	75 (59.06%)
Female	89 (41.98%)	37 (43.53%)	52 (40.94%)
SRE^c^	27 (12.74%)	24 (28.24%)	3 (2.36%)
Anemia	132 (62.26%)	51 (60.00%)	81 (63.78%)
Pathological type			
Adenocarcinoma	182 (85.85%)	69 (81.18%)	113 (88.98%)
SRCC^d^	3 (1.41%)	1 (1.18%)	2 (1.57%)
NETs^e^	17 (8.02%)	8 (9.41%)	9 (7.09%)
Mixed^f^	10 (4.72%)	7 (8.24%)	3 (2.36%)

a Bone metastasis.

b No bone metastasis.

c Skeleton-related events.

d Signet ring cell carcinoma.

e Neuroendocrine tumors.

f Adenocarcinoma mixed with signet ring cell carcinoma.

**FIGURE 1:**
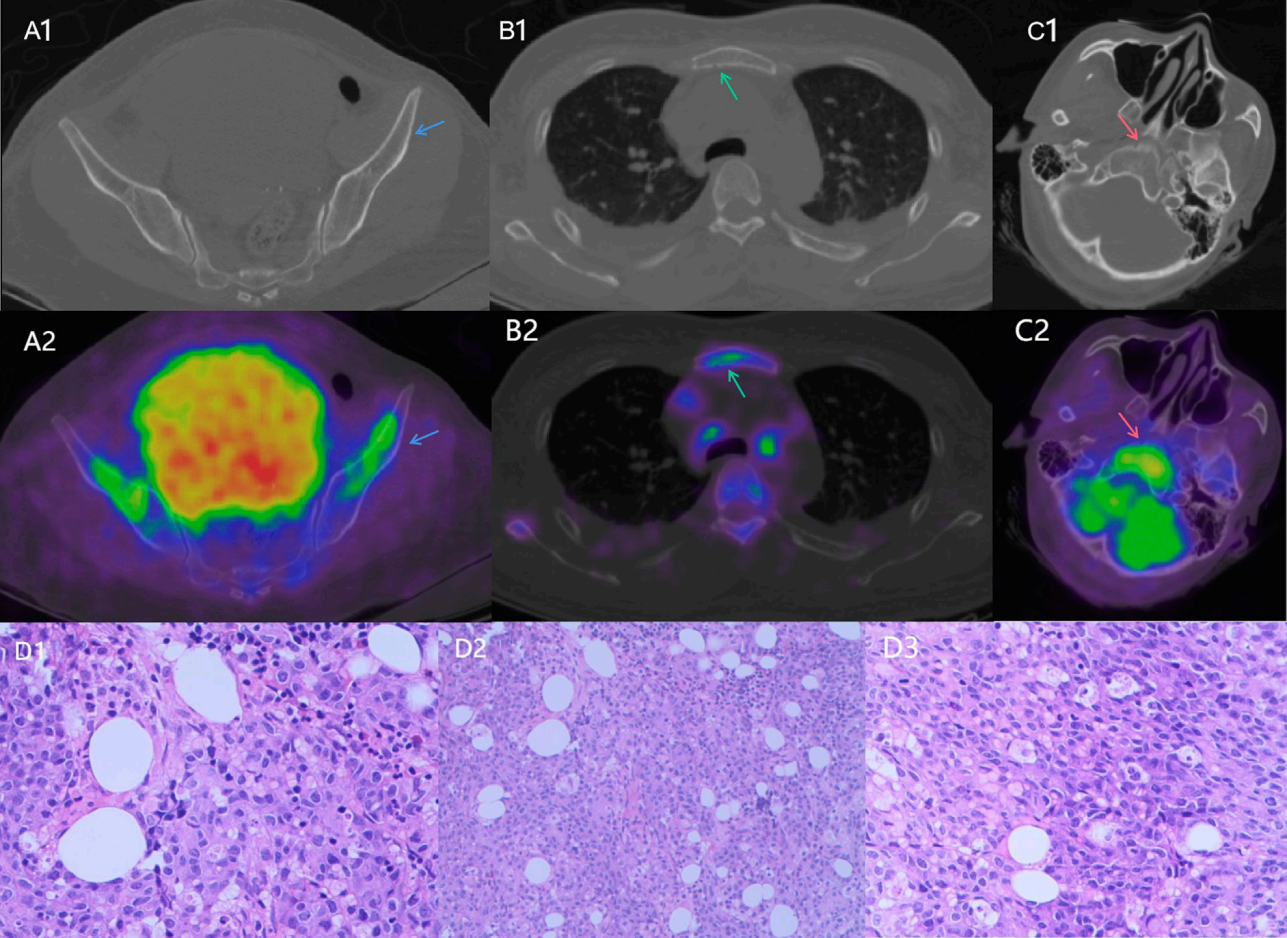
^18^F-FDG PET/CT images of the patient with BM from GC showed extensive osteolytic changes of bone lesions. The axial images A1, B1, and C1 are CT only; A2, B2, and C2 are fused PET/CT. Bone destruction with abnormal FDG uptake (SUVmax = 3.7) was detected in the left ilium (A1, A2, blue arrows), sternum (B1, B2, green arrows) and clival osseous (C1, C2, red arrows). Pathological results of the biopsy of bone lesions showed flaky or focally distributed tumor cells in the bone marrow tissue (D1, D2, D3).

**FIGURE 2:**
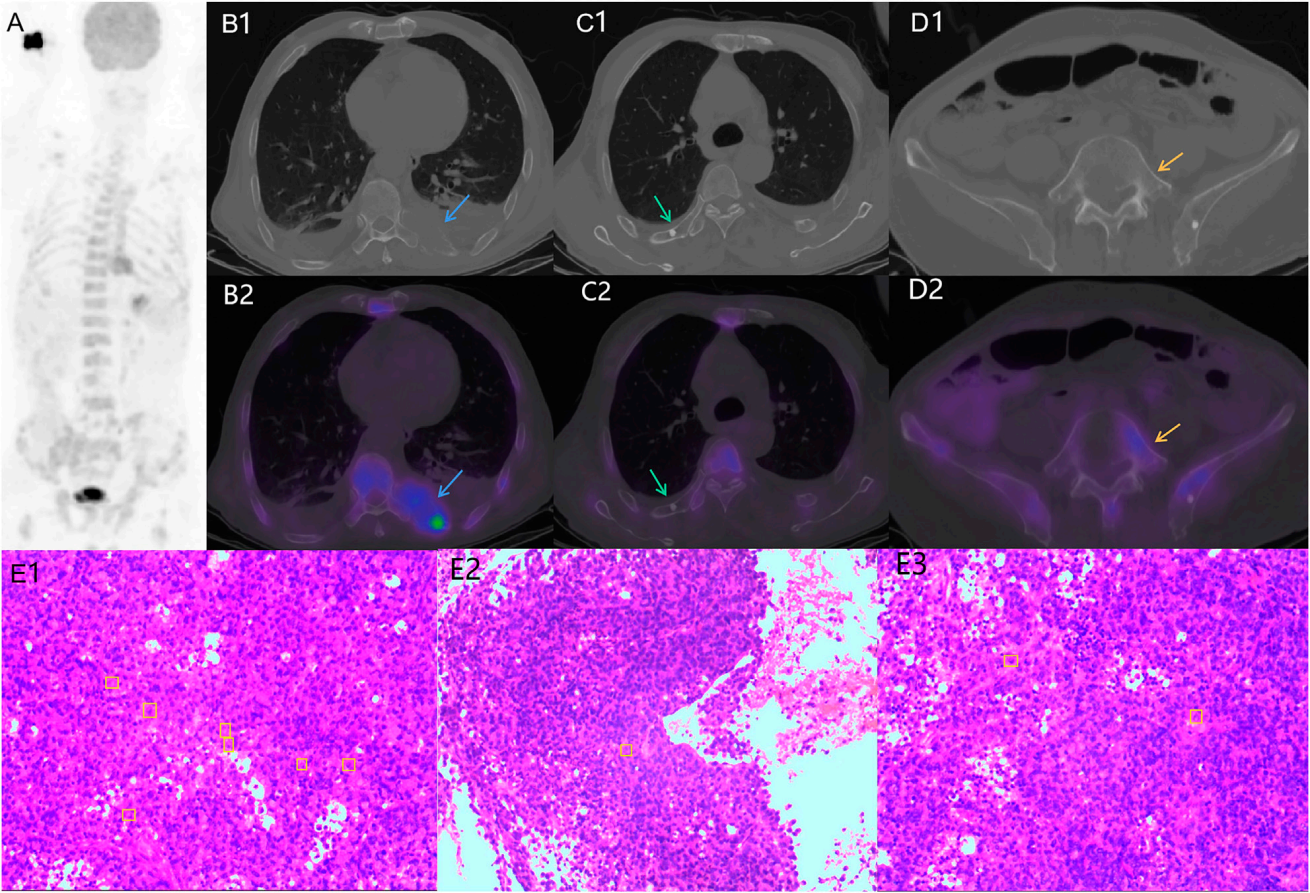
Example ^18^F-FDG PET/CT images of the patient with BM from GC indicated osteogenic changes of bone lesions. **(A)**, maximum intensity projection (MIP); B1, C1, D1, CT only; B2, C2, D2**,** fused PET/CT. Osteogenic changes were seen in the ribs (C1, C2, green arrows) and lumbar vertebrae (D1, D2, orange arrows) with partially abnormal FDG uptake (SUVmax = 12.5). The pathological results (HE staining) of bone lesions (E1, E2, E3) suggested diffusely distributed abnormal cells and some signet ring cells in the tissues.

Among 85 patients with BM, 65 (76.47%) patients had BM at the time of diagnosis of GC(synchronous metastasis). The serum levels of ALP, LDH, CEA, CA19-9, and CA125 were higher in most patients with BM than in patients without BM (Table 2). However, 41.2, 40.0, 49.4, and 49.4% of patients with BM did not show high levels of ALP, LDH, CEA, and CA19-9, respectively (Table 3). Among PET/CT-related factors, the SUVmax of bone lesions in patients with BM (median: 5.60, quartile: 3.20–8.95) was higher than that of patients with no BM (median: 0.00, quartile: 0.00–3.50) (Table 1). The most common bone change was osteolytic bone destruction (42.35%) (Figure 1 and Figures 2B1,B2), followed by osteogenic change (25.88%) (Figures 2C1,C2,D1,D2). Metastatic sites were inclined to be in both axial and appendicular skeletons (64.71%).

**TABLE 2:** Clinical and PET/CT imaging factors.

	Total	BM^a^	NBM^b^
SM^c^/Coexist lesions	135 (63.68%)	65 (76.47%)	70 (55.12%)
ALP ( μ/L)	86 (70,165)	143 (82,356)	77 (66,97)
LDH ( μ/L)	205.00 (167.25,289.50)	300 (195,376)	195 (161,221)
CEA ( μg/L)	3.33 (1.76,9.50)	5.00 (2.16,45.27)	3.00 (1.57,4.69)
CA19-9 ( μ/mL)	16.66 (4.49,102.94)	35.00 (5.56,359.32)	12.35 (4.24,52.65)
CA125 ( μ/mL)	31.50 (13.73,105.28)	46.30 (17.55,136.05)	22.50 (12.20,80.00)
SUVmax of GL^d^	3.75 (1.73,5.80)	3.80 (1.70,6.70)	3.70 (1.80,5.40)
SUVmax of BL^e^	2.90 (0.00 ± 5.60)	5.60 (3.20,8.95)	0.00 (0.00,3.50)
Bone changes			
NSC^f^	104 (49.06%)	16 (18.82%)	88 (69.29%)
OLC^g^	5 0(23.58%)	36 (42.35%)	14 (11.02%)
MC^h^	17 (8.02%)	11 (12.94%)	6 (4.72%)
osteogenic^i^	41 (19.34%)	22 (25.88%)	19 (14.96%)
Bone lesions			
Axial skeletons	99 (46.70%)	24 (28.24%)	75 (59.05%)
appendicular skeletons^j^	18 (8.49%)	6 (7.05%)	12 (9.45%)
Both	95 (44.81%)	55 (64.71%)	40 (31.50%)

a Bone metastasis.

b No bone metastasis.

c Synchronous metastasis.

d Gastric lesion.

e Bone lesion.

f No significant change.

g Osteolytic change.

h Osteolytic change mixed osteogenic change.

i Osteogenic change.

j Appendicular skeletons.

**TABLE 3:** Clinical indicators of BM in GC according to the threshold applied.

Indicators	ALP	LDH	CEA	CA19-9
Clinical threshold	<110 ≥ 110	<240 ≥ 240	<5.00 ≥ 5.00	<35.00 ≥ 35.00
BM in GC	35 (41.2%) 50 (58.8%)	34 (40.0%) 51 (60.0%)	42 (49.4%) 43 (50.6%)	42(49.4%) 43(50.6%)

### Univariate Regression Analysis

**FIGURE 3:**
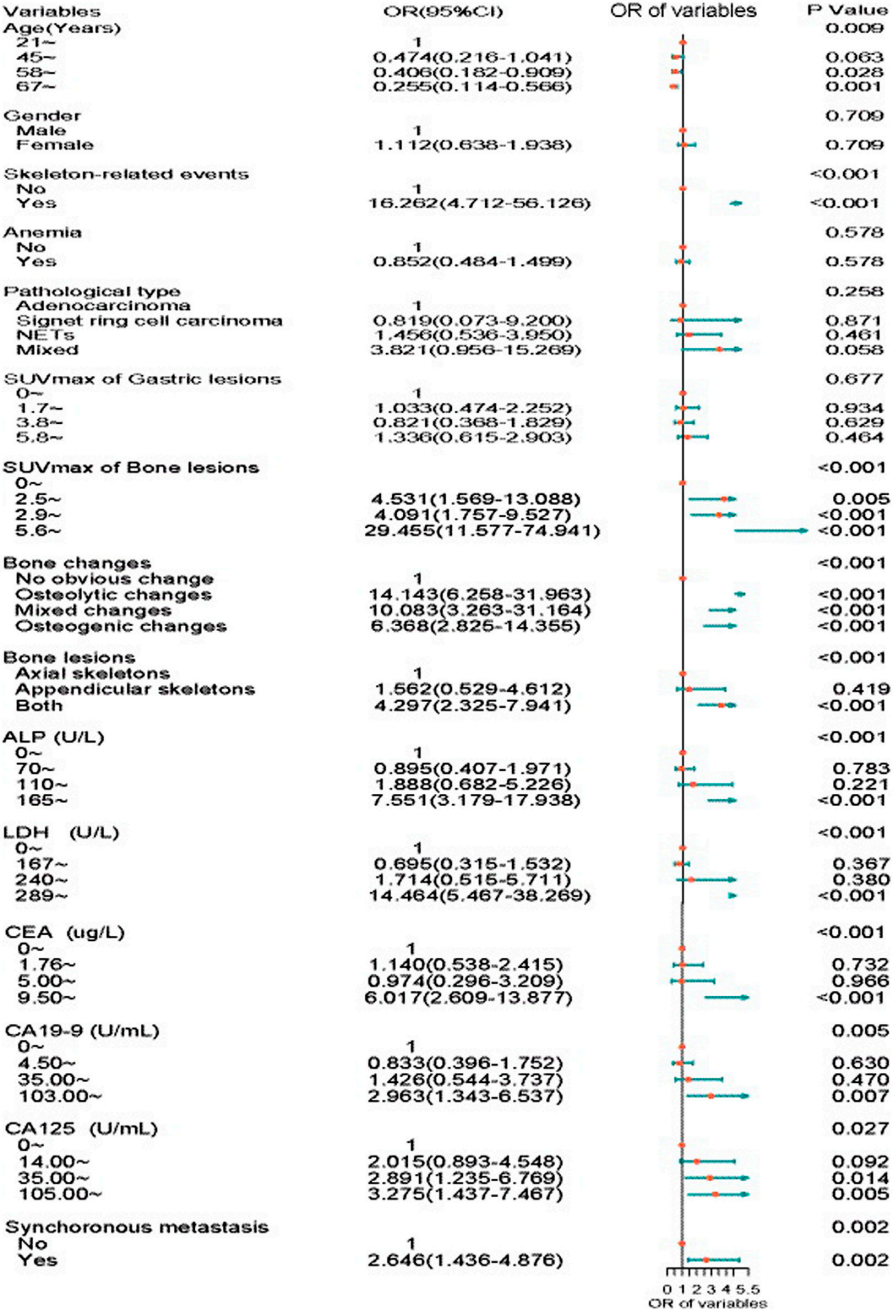
Forest plot summary of univariate logistic regression analysis. Regarding BM as a dependent variable, age, skeleton-related events (SREs), SUVmax of bone lesions, bone changes, location of bone lesions, ALP, LDH, CEA, CA19-9, and CA125 could be recognized as factors for the diagnosis of BM in GC.

The results of the univariate analyses of the diagnostic significance of clinical variables and ^18^F-FDG PET/CT imaging factors concerning the diagnosis of BM are listed in Figure 3. Clinical variables, including age (*p* = 0.009) and SRE (*p* < 0.001), ALP (*p* < 0.001), LDH (*p* < 0.001), CEA (*p* < 0.001), CA19-9 (*p* = 0.005), CA125 (*p* = 0.027), and metastatic pattern (*p* = 0.002), were effective factors for BM, while sex, anemia and pathological type had no effect on BM in GC. The PET/CT imaging factors, including the SUVmax of bone lesions (*p* < 0.001), bone changes (*p* < 0.001), and the location of bone lesions (*p* < 0.001), had diagnostic significance for BM in GC, whereas the SUVmax of gastric lesions did not (Figure 3).

### Statistical Modeling and Validation Demonstrated That the Combination Model had Good Discriminating Power and Predictive Ability

**FIGURE 4:**
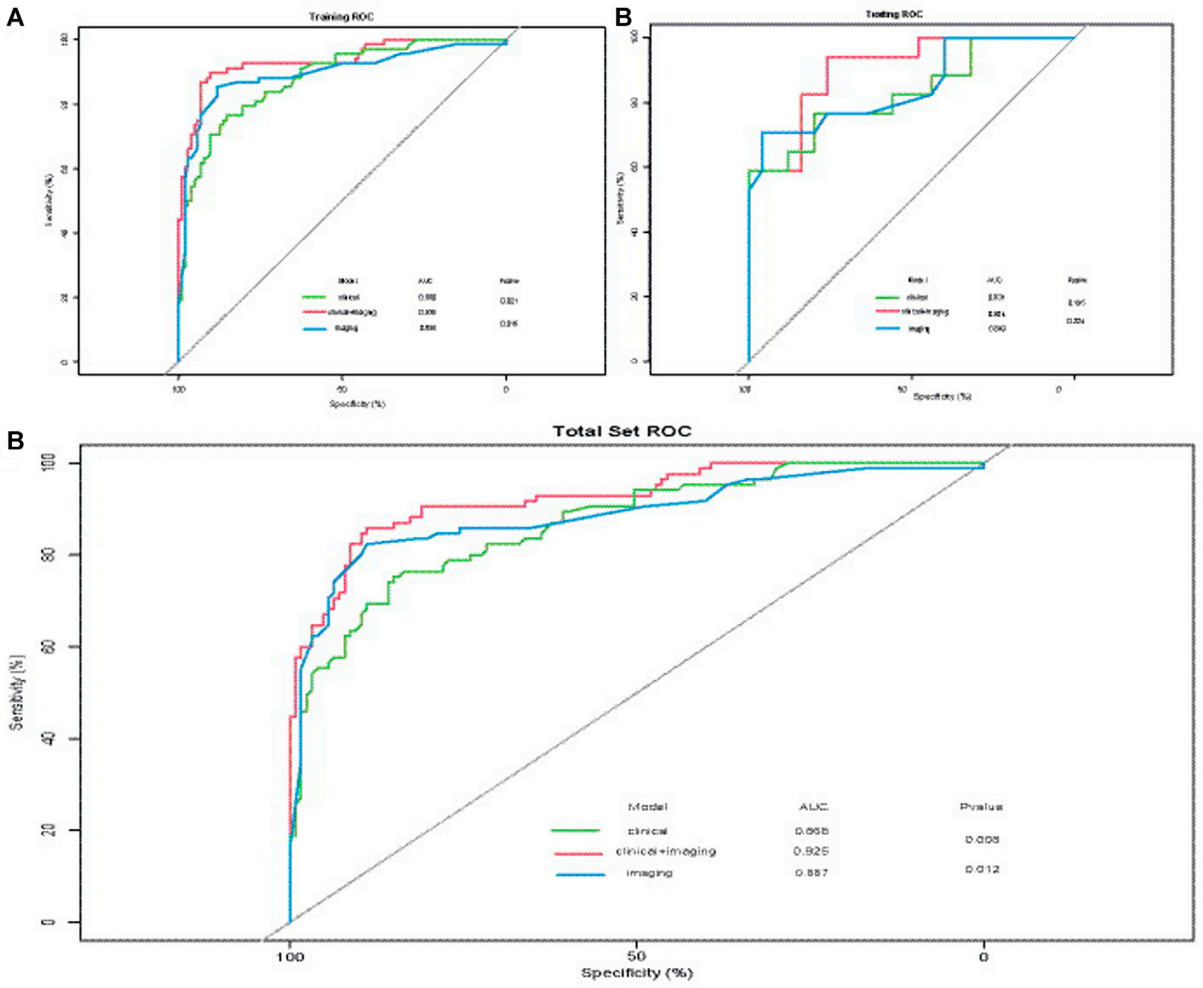
Receiver operating characteristic (ROC) curves were plotted to evaluate the discriminative ability of each model. **(A)** In the training set, the AUC incorporating both clinical and imaging factors was significantly higher than that incorporating only clinical factors or imaging factors (*p* = 0.021, *p* = 0.016, respectively). **(B)** In the validation data, the AUC incorporating both clinical factors and imaging factors was higher than that of the model with clinical factors or imaging factors only, but the difference was not statistically significant. **(C)** In the total set, the AUC of the model including both selected clinical and imaging factors was significantly higher than that including clinical factors or imaging factors only (*p* = 0.008 and *p* = 0.012, respectively).

**FIGURE 5:**
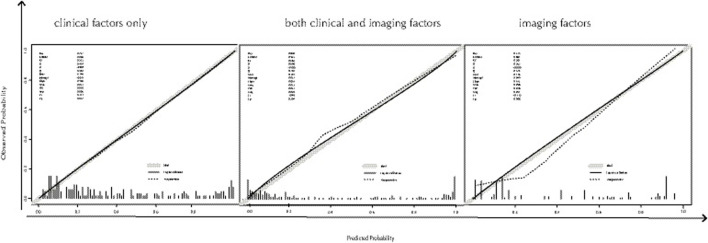
Calibration curves were plotted by using the total set data to evaluate predictive ability that compares observed probabilities with model-predicted probabilities of these three models by using total set data. In the total set, three models that incorporated clinical factors only (on the **left** of Figure 5), both clinical and imaging factors (on the **middle** of Figure 5) and imaging factors only (on the **right** of Figure 5), were well calibrated (*p* = 0.817, *p* = 0.294, *p* = 0.908, respectively).

The AUCs of ^18^F-FDG PET/CT imaging factors, including the SUVmax of bone lesions, bone changes, and location of bone lesions combined with the model generated from clinical factors, including SRE, anemia, ALP, CEA, CA199, and LDH, were significantly higher than those of the model with clinical factors (*p* = 0.021) or ^18^F-FDG PET/CT imaging factors alone (*p* = 0.016) in the training set (Figure 4A). However, there were no statistically significant differences (*p* > 0.05) in the AUCs among these three groups (Figure 4B). The combined model with selected clinical and imaging factors (AUC = 0.904) had a slightly higher AUC than the single-factor model with clinical (AUC = 0.831) or imaging factors alone (AUC = 0.849). Finally, these three models were applied to the total set, including the training set and validation data. The AUC of the combined model with selected clinical and imaging factors (AUC = 0.925) was significantly higher than that of the single-factor model with clinical (AUC = 0.868, *p* = 0.008) or imaging factors alone (AUC = 0.887, *p* = 0.012) (Figure 4C). The calibration plots indicated that all three models would be well calibrated (Figure 5) in the total set.

### Nomogram of This Model May Be Useful in Diagnosing BM in GC

**FIGURE 6:**
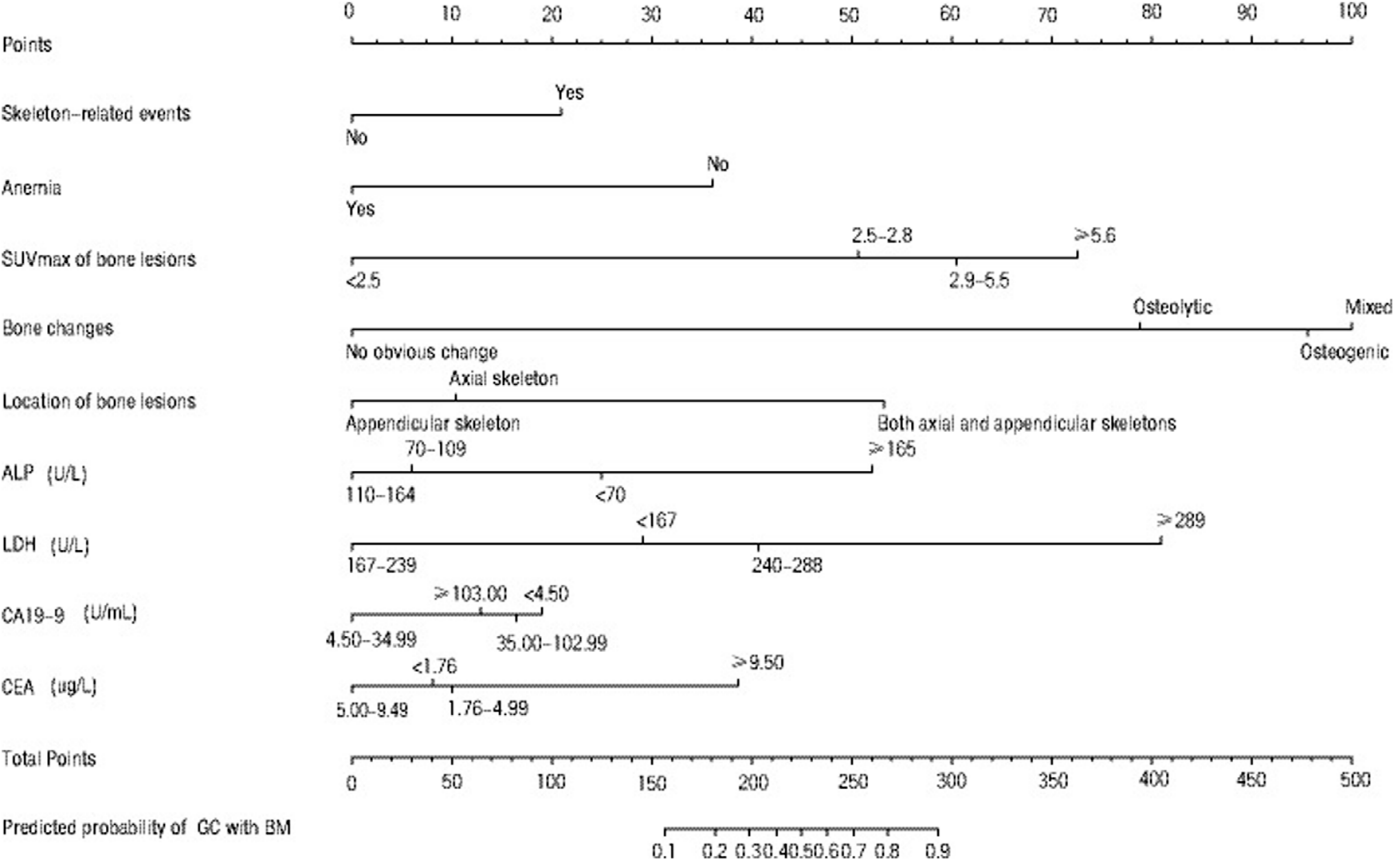
Nomogram for estimating the probability of bone metastasis of gastric cancer. A prognostic nomogram integrating related clinical and imaging factors was generated to diagnose BM from GC. The prediction probability was calculated with reference to Hoshino N et al.16. Mixed indicates osteolytic change mixed with osteogenic change.

We created a nomogram for diagnosing BM in GC based on the binary logistic regression model incorporating both clinical factors and PET/CT-related imaging factors (Figure 6). We prospectively collected three new cases of confirmed BM in GC by pathology after September 2020, and we ran this nomogram for these cases (Table 4). The results suggested that the probabilities calculated by using the nomogram were 60, 90, and 90% and were basically consistent with the actual situation (Table 4).

**TABLE 4:** Points and predicted probabilities calculated by the nomogram for prospective cases from September 2020.

No	SRE^a^/P^b^	Anemia/P	SUV/P	Bone changes/P	Location^b^/P	ALP/P	LDH/P	CA19-9/P	CEA/P	Total points	Predicted probability^c^	Pathological results
1	No/0	Yes/0	9.9/72.5	Osteolytic/77.5	Both/52.5	66/25	172/0	24.54/0	3.15/10	237.5	Approximately 60%	BM^d^ in GC^e^
2	Yes/20	Yes/0	8.2/72.5	Osteogenic/95	Both/52.5	900/50	1319/80	299.9/13	107.04/37.5	383	More than 90%	BM in GC
3	No/0	Yes/0	8.4/72.5	Osteogenic/95	Both/52.5	239/50	/80	-	13.41/38	388	More than 90%	BM in GC

a Skeleton-related events; ^b^points.

b Location of bone lesions.

d Bone metastasis.

e Gastric cancer.

c Predicted probability of suffering from BM in GC.

## Discussion

The capability for early diagnosis has increased for some common metastases of GC, such as lung, liver and brain (Qiu et al., 2018; Johnston and Beckman, 2019), but the early diagnosis of BM in GC still requires further study. It is important to confirm BM for management and treatment of patients with GC. However, a suitable model for the accurate diagnosis of bone metastases in gastric cancer patients is still not available. In this study, we incorporated PET/CT imaging findings, including the SUVmax of bone lesions, bone changes, and the location of bone lesions, with clinical data, including SRE, anemia, ALP, LDH, CA19-9, and CEA, into the binary logistic regression model. The verification of this model suggested the good calibration ability and a better distinguishing ability of this combined model compared with the model that included only PET/CT-related data or clinical data. Nomograms are commonly used tools to estimate diagnosis and prognosis in oncological and medical studies and can generate an individual numerical probability of a clinical event by integrating diverse determinant variables (Balachandran et al., 2015). We generated a suitable nomogram for accurately indicating BM in GC patients. Our research aimed to explore an effective diagnostic model that combined ^18^F-FDG PET/CT imaging findings and clinical factors to assess BM in GC.

Yao, G et al. reported that the SUVmax was a promising and valuable metabolic indicator for the prediction of metastasis from FDG-avid bone lesions indicated by ^18^F-FDG PET/CT (Yao et al., 2020). Most of the studies set the SUVmax threshold between 2 and 4.3 (Yao et al., 2020). Conies et al. reported that an SUVmax >4 indicated a malignant lesion (Cornelis et al., 2014), which was consistent with our study. SUVmax is a semi-quantitative index referring to radioactive uptake in the lesion and represents cell proliferation activity. It has also been reported that there were abnormal uptake in some bone lesions, while the structure of bones seemed to be normal (Al-Muqbel, 2017). Therefore, SUVmax is a very important indicator for predicting bone metastasis.

Our study suggested that the most common bone change in BM from GC was the osteolytic type, which is consistent with previous studies (Silvestris et al., 2013; Imura et al., 2020). Moreover, in previous reports, BM of GC most commonly occurred in axial skeletons (Wen et al., 2019; Imura et al., 2020); whereas, Vestris, N et al. suggested that patients showed the majority of metastatic sites in appendicular skeletons, followed by hip (38%) and spine (Silvestris et al., 2013). However, our research indicated that both axial skeleton and appendicular skeleton lesions were most commonly present in BM from GC. We believe that most patients with gastric cancer show symptoms of anemia, leading to active hematopoiesis, which increases the radioactive uptake of axial skeletons. Therefore, false positive results could be due to the abnormal uptake of axial bone. Then, the changes in both axial skeletons and appendicular skeletons are more likely due to metastatic lesions.

ALP is specifically expressed on the surface of osteoblasts. Osteoblast and osteoblastic precursor activity can be represented by the serum level of bone ALP (Moss, 1987). Our research suggested that abnormally elevated ALP was a risk factor for gastric cancer with bone metastasis. A serum level of ALP ≥165 U/L could be interpreted as a sign of BM in GC patients. We reasoned that cancer cells metastasizing from GCs to bones activated osteoblasts and osteoclasts, which generated ALP. However, the serum levels of ALP were not always increased in patients with metastasis; therefore, it was necessary to analyze a combination of other markers, including LDH, CEA, and CA19-9. Serum levels of LDH, CEA, and CA19-9 are prognostic factors that assist in the diagnosis and survival prediction of patients with BM from GC (Kobayashi et al., 2005; Turkoz et al., 2014; Mikami et al., 2017). Furthermore, our study suggested that when LDH ≥289 μ/L, CEA≥9.5 μg/L, and CA19-9 ≥35.0 μ/mL, the risk of bone metastasis in patients with gastric cancer increased significantly. LDH catalyzes the reversible process of pyruvate to lactate under anaerobic conditions and promotes the production of lactate, which induces the proliferation of oxygenated malignant cells and angiogenesis and inhibits innate and adaptive immune responses (Deme and Telekes, 2017). As a result, we believe LDH would be one of the biomarkers to assist in the diagnosis of metastatic lesions. Although CEA and CA19-9 were used as markers of gastrointestinal malignancies (Bagaria et al., 2013; Gao et al., 2018; Jia et al., 2019), in our study, the serum levels of CEA and CA19-9 in GC patients without BM did not increase significantly. The serum levels of CEA and CA19-9 in gastric cancer patients in the BM group were slightly increased. This result suggests that CEA and CA19-9 were not necessarily elevated in gastrointestinal patients but increased in patients with bone metastasis in gastrointestinal cancer. Therefore, we speculated that CEA and CA19-9 played an important role in confirming BM in patients with GC, which was consistent with Tsukushi’s study (Tsukushi et al., 2006). Moreover, nearly half of patients who had confirmed BM in GC had serum levels of ALP, LDH, CEA, and CA19-9 lower than the threshold for clinical application in our present study. Therefore, it was necessary to combine ^18^F-FDG PET/CT with other clinical factors for the diagnosis of BM in GC.

Our results and Qiu’s studies (Qiu et al., 2018) showed that patients with bone metastasis were approximately 7 years younger than those without bone metastasis. Additionally, our results also indicated that pathological types of GC were not a risk factor for BM in GC. Although age, CA125 levels and metastatic patterns were statistically significant in a single-factor analysis, we did not include them in our analytic model because the increased misclassification rate due to the sample size was not optimal. In addition, the development of statistical models in external validation was hampered by the small number of subjects per group, resulting in no difference in the AUC of the three models and poor calibration in the combined model with both clinical and imaging factors.

Our nomogram incorporating clinical and ^18^F-FDG PET/CT imaging factors could provide nuclear medicine physicians and clinicians information regarding the precise probability of BM in GC. The symptoms of suspicious bone lesions in patients with a history of GC or bone lesions and suspicious bone lesions and gastric lesions simultaneously by ^18^F-FDG PET/CT could alert physicians to monitor clinical indicators, including ALP, LDH, CEA and CA19-9 levels, for the diagnosis of BM and thus for the development of a precise treatment plan.

There were several limitations in our study. First, this was a retrospective study. Insufficient information as a result of many lost cases resulted in unsatisfactory sample sizes. In addition, pathological examinations as the gold standard for diagnosing BM in patients with GC were only conducted in a few cases.

In conclusion, a diagnostic model that combines ^18^F-FDG PET/CT-related imaging findings, such as the SUVmax of bone lesions, bone changes, and the location of bone lesions, with clinical data, including skeleton-related events, anemia, ALP, LDH, CA19-9, and CEA, might assist in the diagnosis of GC with BM. Our nomogram could be helpful for assessing the risk of bone metastasis of gastric cancer.

## Data Availability

The original contributions presented in the study are included in the article, further inquiries can be directed to the corresponding authors.
